# 18F-FDG PET/CT Staging of Gastroesophageal Junction Adenocarcinoma With Skeletal Muscle Metastasis: A Case Report

**DOI:** 10.7759/cureus.102847

**Published:** 2026-02-02

**Authors:** David Gutierrez Albenda, Ana María Gutiérrez, Gabriel Infante, Mariana Parra, Ian Taylor

**Affiliations:** 1 Cyclotron-PET/CT Laboratory, Universidad de Costa Rica, San José, CRI; 2 School of Medicine, Universidad de Costa Rica, San José, CRI

**Keywords:** 18f-fdg radiotracer, cancer staging, esophageal cancer, gastroesophageal junction adenocarcinoma, pectoralis major metastasis, triceps brachii metastasis

## Abstract

Malignancies of the upper digestive tract, including esophageal, gastroesophageal junction (GEJ), and gastric carcinoma, tend to present clinically with advanced pathologic staging. This has led to much debate regarding optimal imaging modalities for the purpose of disease staging.

We present the case of a 54-year-old patient with a history of gastroesophageal reflux disease (GERD), who presented with a Siewert Type III GEJ adenocarcinoma with symptomatic skeletal muscle (SM) metastasis. On ^18^F-fluorodeoxyglucose (^18^F-FDG) positron emission tomography with computed tomography (PET/CT), the patient was found to have multiple lymph nodes, bone, and SM metastases. This case report also provides a brief review of the current literature regarding ^18^F-FDG PET/CT use for esophageal and GEJ carcinoma pathologic staging.

## Introduction

Esophageal and gastric carcinomas are an ever-increasing cause of morbidity and mortality worldwide [1]. Gastroesophageal junction (GEJ) adenocarcinomas in particular pose a therapeutic and surgical challenge when it comes to determining the optimal treatment; depending on the epicenter of the tumor, they tend to behave either as esophageal or gastric carcinomas. The adequate approach to the staging of these malignancies remains a subject of much debate in the medical literature [2-6]. Even more so, the subject of appropriate imaging modalities for posttreatment staging of the disease has yet to be properly studied.

We report the case of a 54-year-old male who presented with Siewert Type III GEJ adenocarcinoma with skeletal muscle (SM) metastases, who underwent ^18^F-fluorodeoxyglucose (^18^F-FDG) positron emission tomography with computed tomography (PET/CT) for posttreatment staging of the disease.

## Case presentation

A 54-year-old male with a history of poorly controlled gastroesophageal reflux disease (GERD) underwent upper endoscopy for evaluation of persistent symptoms. He was otherwise healthy. His medical history included a 10-pack-year smoking history, having quit 20 years prior, and regular alcohol consumption.

Upper endoscopy revealed an exophytic mass located 2-3 cm distal to the GEJ. Histopathological analysis of biopsy samples demonstrated a moderately differentiated adenocarcinoma. Initial contrast-enhanced CT staging classified the disease as T2N1M0. The patient was started on combined chemoradiotherapy, followed by total gastrectomy. Postoperative CT imaging showed no residual tumor.

Several days after surgery, the patient noted a palpable mass in the right pectoral region. Biopsy of the lesion revealed metastatic infiltration by moderately differentiated adenocarcinoma. Subsequently, FDG-PET/CT was performed for further staging. The study was performed using a Siemens Biograph Vision 450 Edge (Flow) system (Siemens Healthineers, Erlangen, Germany). It consisted of a high-spatial-resolution PET acquisition using lutetium-yttrium oxyorthosilicate (LYSO) detectors, with axial-plane slices obtained at a table speed of 1 mm/s. PET image reconstruction was carried out using the TrueX + Time-of-Flight (ultraHD-PET) algorithm, with two iterations and five subsets, a zoom factor of 1, a 5.0-mm Gaussian filter, and attenuation correction.

The PET study was combined with a low-dose CT scan using CareDOSE technology. CT acquisition was performed in a helical, 128-slice, multidetector mode, with 5-mm slices and a pitch of 0.6. CT reconstruction was performed with 2-mm slices using the SAFIRE iterative reconstruction method, a Body Regular (BR34) reconstruction filter, and a fast mediastinal window. The field of view was 500 mm, with 1-mm slice increments and an intensity level of 3. The study was performed without intravenous contrast. The patient’s blood glucose level prior to administration of ^18^F-FDG was 89 mg/dL. A dose of 9.9 mCi of ^18^F-FDG was administered intravenously through the left upper limb. Image acquisition began 60 minutes after radiotracer administration. The acquisition protocol extended from the cranial vertex to the proximal third of the thighs. No additional images were obtained.

**Figure 1:**
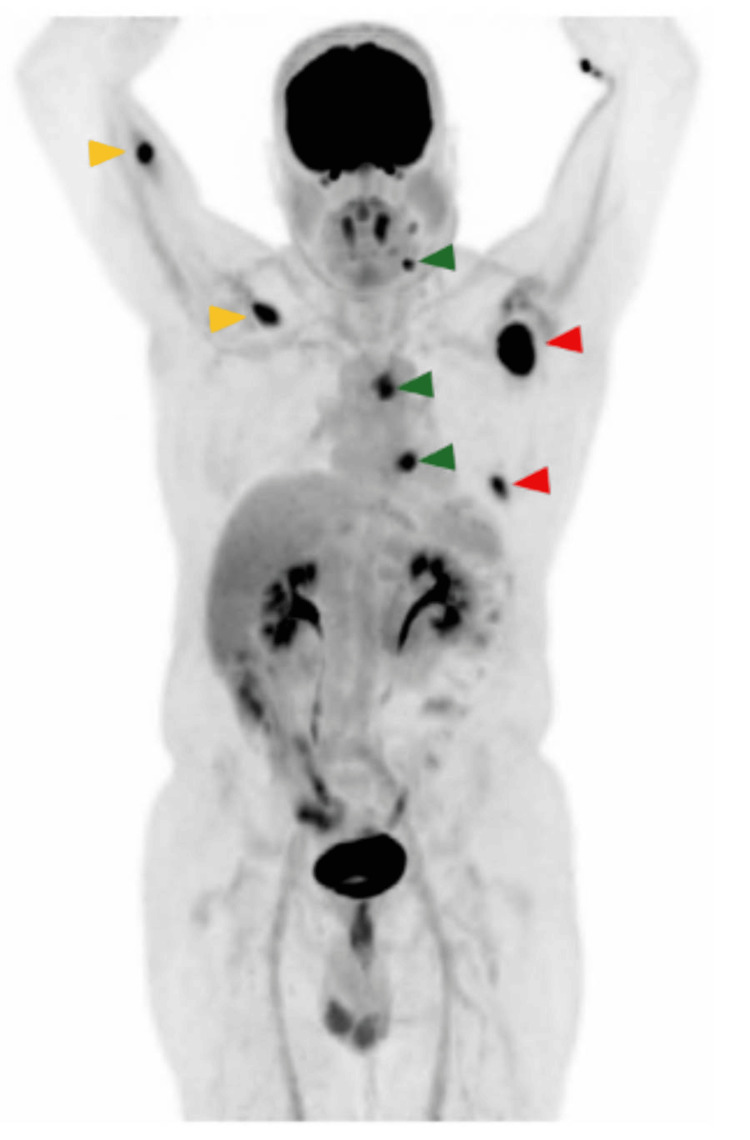
MIP image from 18F-FDG PET/CT The study reveals hypermetabolic cervical and mediastinal lymph nodes (green arrowheads), skeletal muscle metastases involving the right pectoralis major and triceps brachii muscles (yellow arrowheads), and osseous metastatic lesions (red arrowheads), consistent with disseminated metastatic disease. MIP: maximum intensity projection; ^18^F-FDG PET/CT: ^18^F-fluorodeoxyglucose

**Figure 2:**
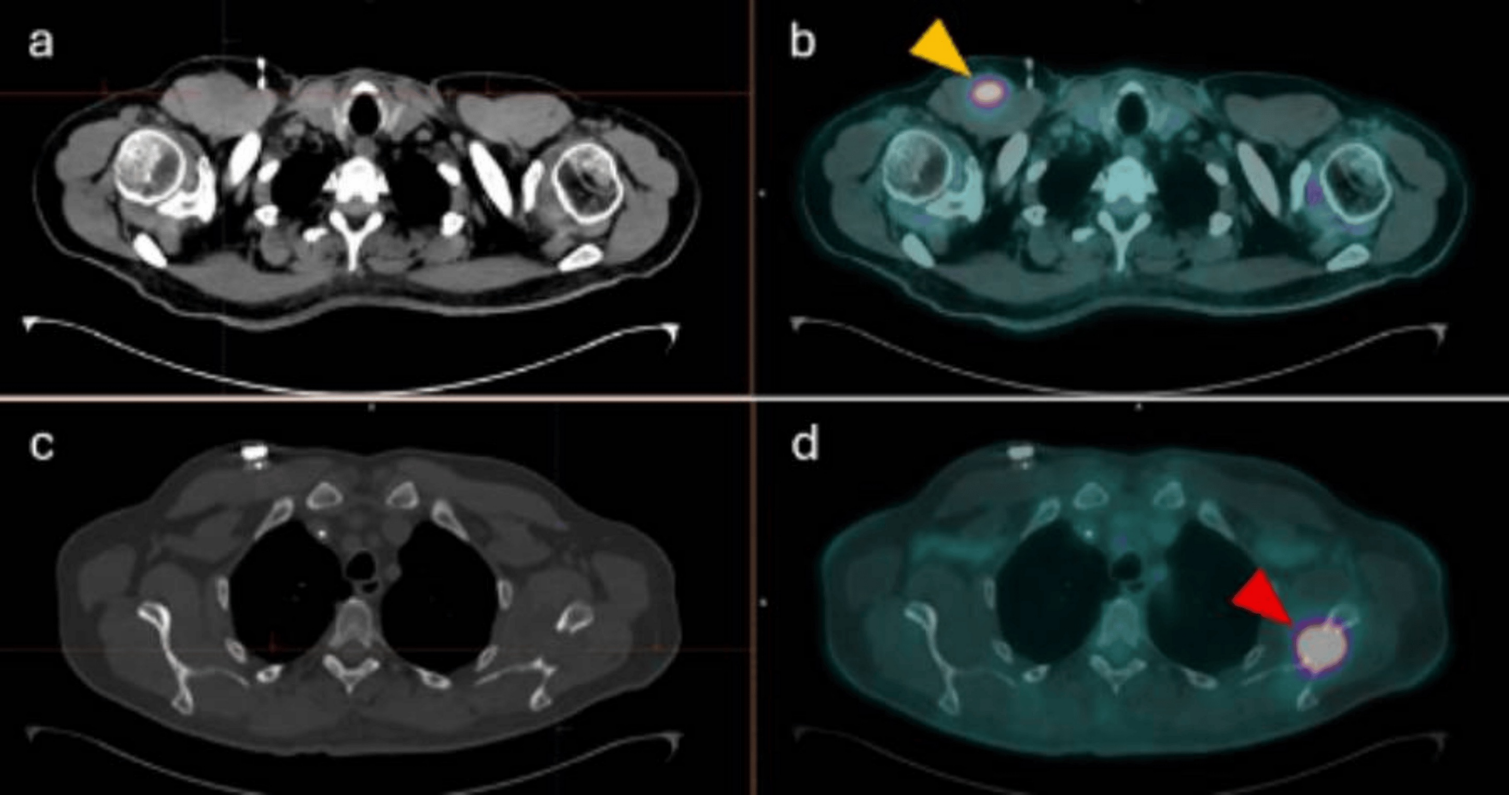
Axial CT and axial 18F-FDG PET/CT Images (a) and (b) demonstrate a hypodense, hypermetabolic lesion involving the right pectoralis major muscle (yellow arrowhead), consistent with skeletal muscle metastasis. Images (c) and (d) demonstrate a hypermetabolic, expansile, osteolytic lesion with an associated soft-tissue component involving the neck of the left scapula (red arrowhead), consistent with osseous metastatic disease. ^18^F-FDG PET/CT: ^18^F-fluorodeoxyglucose positron emission tomography with computed tomography

The study demonstrated multiple hypermetabolic lesions, including left cervical lymph nodes (levels Ib and IIa), left periesophageal and hilar lymph nodes, as well as nodular hypermetabolic lesions within the right pectoralis major and triceps brachii muscles (Figures 1, 2). Additionally, expansive osteolytic lesions with intense FDG uptake were identified in the posterolateral aspect of the seventh rib and the neck of the left scapula, the latter showing cortical disruption with extension into the supraspinatus, infraspinatus, and subscapularis muscles, suggesting direct infiltration. A small volume of free fluid was also observed in the pelvic cavity, suspicious for malignant ascites.

## Discussion

Current epidemiology and classifications of GEJ cancer

Gastroesophageal cancers are an important cause of morbidity and mortality worldwide. According to the International Agency for Research on Cancer (IARC), as of 2022, stomach cancer and esophageal cancer are currently the fifth and seventh leading causes of cancer-related mortality. The combined estimates for both of these conditions surpass 1 million related deaths [1]. Both conditions also have important racial disparities in incidence, mortality, and survival rates across social and racial lines [7]. 

The Siewert classification classifies GEJ carcinomas with regard to the endoscopic location of the lesion and its relation to the GEJ. Siewert Type 1 GEJ tumors are located 1-5 cm proximal to the anatomic GEJ; Type 2 tumors may span or cross the GEJ, and their epicenter may be located at most 2 cm distal to the GEJ; Type 3 tumors will span 2-5 cm into the stomach [8-10]. Siewert Types 1 and 2 tumors are treated as esophageal cancer and require only partial gastrectomy; meanwhile, Type 3 tumors (like the one presented in our patient) are treated as gastric cancer, with total gastrectomy indicated [8]. The clinical importance of this classification is noted in the fact that Type 3 tumors have been documented to present at later stages and have worse outcomes following surgery than patients with Types 1 and 2 GEJ carcinomas.

SM metastasis in adenocarcinoma of the GEJ

Of note, our patient presents with SM metastasis, which is a rare occurrence in GEJ cancers. A case report published by Sabu et al. [8] provided a comprehensive literature review of documented cases of SM metastasis in GEJ adenocarcinoma. No pectoralis major or triceps brachii metastases, such as the ones presented in our patient, are detailed. SM metastases are rare due to poor adherence of tumor cells caused by varying perfusion, and because myocytes produce leukemia inhibitory factor and interleukin-6, molecules with anticancer properties [11].

PET/CT imaging is usually critical in the detection of these lesions because, unlike in our patient, SM metastases tend to be asymptomatic [8]. SM metastases are usually an incidental finding on these studies. Other imaging modalities, such as CT scans, have proven to be inadequate in comparison to PET/CT when diagnosing SM metastases [12,13].

Current evidence regarding the use of ^18^F-FDG PET/CT staging in esophageal cancers

There are a number of studies that have sought to determine the utility of ^18^F-FDG PET/CT for staging esophageal carcinomas, particularly when compared to other imaging modalities. Giganti et al. [2] compared the diagnostic performance of PET/CT in preoperative locoregional staging of esophageal cancer against magnetic resonance (MR) and diffusion-weighted imaging (DWI), multidetector CT, and endoscopic ultrasonography (EUS); the enrolled patients had either esophageal or Siewert Type 1 tumors. PET/CT showed lower sensitivity and specificity for T and N staging when compared to EUS or MR with DWI. Notably, the sample size was small, with only 18 patients included in the study. 

Some studies compare ^18^F-FDG PET and CT scans separately regarding their staging capabilities. van Vliet et al. [3] published a meta-analysis comparing the diagnostic performance of EUS, CT, and ^18^F-FDG PET in the staging of esophageal cancers. Diagnostic performance for regional lymph node metastasis did not differ significantly between the three tests; EUS did prove to have the highest sensitivity in this regard. ^18^F-FDG PET was shown to have significantly higher diagnostic performance for distant metastases than CT. Other studies similarly concluded that ^18^F-FDG PET appears to have a better capability of detecting organ metastases when compared to CT alone [4,5], while also noting that the diagnostic value of PET in the staging of adenocarcinomas of the esophagus and GEJ is limited due to low accuracy in the staging of paratumoral and distant lymph nodes. 

Most of the studies regarding esophageal carcinoma staging currently available in the medical literature compare the different study modalities in the pretreatment staging of esophageal or GEJ adenocarcinomas. There are some studies that have delved into the importance of posttherapy pathologic staging as a marker of disease-free and overall survival in these patients, without comparing different imaging modalities in this regard [6]. No studies reviewed for this case presentation compared imaging modalities for staging among different Siewert-type GEJ tumors.

## Conclusions

We report a rare case of Siewert Type III GEJ adenocarcinoma with symptomatic SM metastases detected on FDG-PET/CT during posttreatment staging. This case highlights the value of FDG-PET/CT in identifying distant and atypical metastatic sites that may be missed on conventional imaging. Although further studies are needed to better define its role in posttreatment staging of GEJ adenocarcinoma, FDG-PET/CT appears to enhance the detection of organ metastases and may contribute to improved clinical decision-making.
